# Delayed Histiocytic Foreign Body Reaction and Granuloma Formation Following Argiform Injection for Soft Tissue Augmentation Mimicking a Lipomatous Tumor on Histopathology: A Case Report

**DOI:** 10.1155/crdm/7330965

**Published:** 2026-10-19

**Authors:** Mokhtar H. Abdelhammed, Lois M. Dodson, A. Hafeez Diwan, Ya Xu

**Affiliations:** ^1^ Department of Pathology & Immunology Baylor College of Medicine Houston 77030 Texas USA bcm.edu; ^2^ Department of Dermatology Baylor College of Medicine Houston 77030 Texas USA bcm.edu; ^3^ Department of Pathology & Laboratory Medicine Harris Health System Ben Taub Hospital Houston 77030 Texas USA; ^4^ Department of Surgery Pathology Service HCA Houston Healthcare Clear Lake Webster 77598 Texas USA

**Keywords:** Argiform, augmentation, foreign body, granuloma, histiocytic

## Abstract

As cosmetic procedures gain popularity, the use of nonapproved soft‐tissue fillers by unlicensed or untrained practitioners in nonmedical settings is rising. We report a rare case of a histiocytic reaction to foreign material following Argiform injection. A 31‐year‐old female presented with a 2 year history of an itchy and painful rash on both buttocks. The rash was initially confined to the right buttock but extended to the left several months later; it remained refractory to topical ointments. A skin punch biopsy revealed extensive adipocytic‐like cells within the dermis, some of which mimicked lipoblasts. Immunohistochemistry showed the lesional cells were positive for CD68 and negative for S100 and pancytokeratin. A final diagnosis of a histiocytic reaction to foreign material was made. Following the biopsy results, the patient disclosed a history of cosmetic Argiform injections at a spa 4‐5 years prior to presentation. This case demonstrates that delayed tissue reactions to Argiform can mimic lipomatous tumors, creating a significant diagnostic pitfall. Clinicopathologic correlation, including a thorough remote medical history, is critical for achieving an accurate diagnosis.

## 1. Introduction

With the increasing demand for cosmetic procedures and aesthetic rejuvenation, the widespread use of nonapproved tissue expanders by unlicensed or untrained practitioners in nonmedical settings continues to rise. Several fillers and tissue‐augmenting agents have been developed in recent decades. These can be broadly classified based on human enzymatic elimination into resorbable (biodegradable) agents, which commonly include collagen of various sources, hyaluronic acid (HA), poly‐L‐lactic acid (PLLA), and calcium hydroxylapatite, and permanent (nonbiodegradable) agents, which include paraffin, silicone, hydroxyethyl methacrylate, polymethylmethacrylate (PMMA), and polyacrylamide hydrogel (PAAG) [1, 2].

Argiform is a permanent PAAG filler that is not US‐FDA approved [3]. Currently, PMMA/Bellafill remains the single FDA‐approved permanent soft‐tissue filler, with indications strictly limited to nasolabial folds and cheek acne scars [3]. Nonpermanent or permanent fillers for breast and buttock augmentation, glabellar injections, and foot fullness remain strictly unapproved [3]. We report a rare case of histiocytic reaction to foreign material after Argiform (a hydrophilic polyacrylamide gel product) injection for buttock augmentation.

## 2. Case Report

A 31‐year‐old female presented with bilateral rashes on her buttocks that had persisted for 2 years. Initially, the rash was confined to the right buttock but extended to the left side several months later; it was associated with itchiness and pain. The patient denied any systemic symptoms such as fever, chills, night sweats, or unintentional weight loss. On examination, a 4.0 cm firm, skin‐colored indurated plaque on the right buttock progressively expanded medially across the gluteal cleft to involve the left buttock. Since no distinct subcutaneous nodules or deep palpable masses were identified in the bilateral buttocks, the clinical suspicion for a deep soft‐tissue neoplasm was low, and no imaging study (such as ultrasound or magnetic resonance imaging) was performed. Despite the use of triamcinolone and hydrocortisone topical ointments, her symptoms persisted. Because the lesion was refractory to treatment and the medical history was initially unclear, a punch biopsy was performed.

Grossly, our punch biopsy specimen measured 0.4 × 0.4 cm and was excised to a depth of 1 cm. Microscopically, the skin punch biopsy showed the dermis with extensive adipocytic‐like cells. The lesional cells were variable in size, with some mimicking multivacuolated lipoblasts or signet ring‐like, univacuolated lipoblasts (Figure 1). Multinucleated foreign body giant cells and extracellular filler material were not identified. A differential diagnosis of atypical lipomatous tumor (ALT), carcinoma with signet ring cell features, and infection was considered. Immunohistochemistry (IHC) was performed; the adipocytic‐like cells were positive for CD68 and negative for S100 and Pancytokeratin (Figure 2). No acid‐fast bacilli (AFB) or fungal organisms were identified by AFB and Grocott‐Gomori methenamine silver (GMS) special stains, respectively. The final diagnosis was rendered as “histiocytic reaction to foreign material,” with a comment noting that “this can be seen in silicone granuloma.”

**FIGURE 1:**
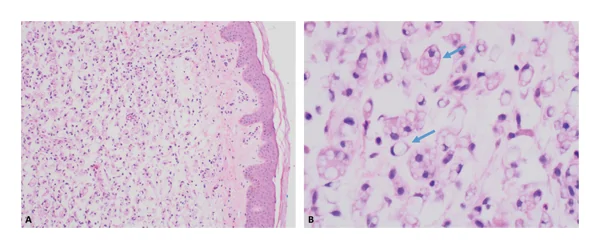
Histological features of the skin punch biopsy from the right buttock (hematoxylin and eosin, H&E). (A) The dermis exhibits extensive adipocytic‐like vacuolation of variable sizes (40*x*). (B) Higher magnification (400*x*) reveals cells mimicking multivacuolated lipoblast (upper arrow) and univacuolated (signet‐ring‐like) lipoblast (lower arrow).

**FIGURE 2:**
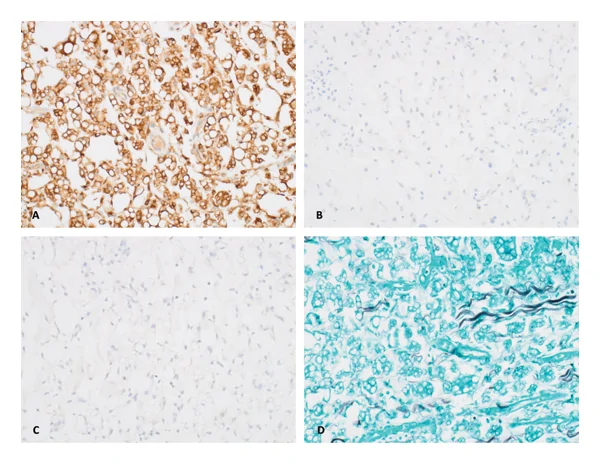
Immunohistochemical and special stain findings (200*x*). (A) The adipocyte‐like cells display strong positivity for CD68, confirming their histiocytic origin. The cells are negative for (B) pancytokeratin and (C) S100, ruling out metastatic carcinoma and adipocytic neoplasia, respectively. (D) GMS special stain is negative for fungal organisms, showing only nonspecific background staining of the fibrous tissue.

Histopathological findings, which included a histiocytic reaction to foreign material, prompted further clinical history. Subsequently, the patient reported receiving cosmetic Argiform injections at a local spa center 4‐5 years prior to her presentation.

The patient was managed conservatively with a single intralesional corticosteroid injection (1.0 mL of triamcinolone acetonide [Kenalog], 10 mg/mL) into the superior right buttock, rather than undergoing systemic therapy or surgical excision, and tolerated the procedure well. Although serial clinic appointments were scheduled to evaluate symptomatic improvement and monitor her response to treatment, she subsequently failed to return and was lost to follow‐up. Consequently, long‐term outcome data and symptom resolution could not be assessed.

## 3. Discussion

The ideal soft‐tissue filler should achieve a predictable aesthetic result while maintaining a high safety profile, host biocompatibility, and minimal risk of late‐onset migration or inflammatory sequelae [4, 5]. However, many commonly used injectables are associated with various adverse events. Acute complications often include localized inflammatory reactions, infections, sterile abscesses, and hypersensitivity. Conversely, permanent agents are frequently linked to long‐term complications such as chronic granuloma formation, distant material migration, tissue necrosis, and persistent scarring [1, 6, 7]. While biodegradable agents typically resolve within their respective half‐lives, permanent fillers, such as PAAGs, often produce effects that are refractory to conservative treatment and may necessitate surgical intervention [1, 8].

A significant clinical challenge in diagnosing PAAG complications is navigating the variable latency period between injection and symptom onset. Christensen et al. [7] evaluated tissue responses across permanent dermal fillers, noting that while unreactive PAAG (Aquamid) displays minimal host reaction, adverse events present as inflammatory nodules driven by foreign body responses, typically emerging within the first year postinjection. In contrast, Ono et al. [8] documented broader long‐term complications following PAAG (Amazingel) administration for soft tissue augmentation, observing that while the material is often marketed as a stable agent, it frequently leads to delayed nodule formation, induration, migration, and chronic inflammatory sequelae years after implantation. Notably, detailed clinical reports focusing specifically on Argiform complications are not currently found in indexed databases. Our case directly mirrors these extended temporal observations, as the patient presented with firm, refractory plaques in the buttocks several years after the initial procedure. This timeline reinforces the necessity of maintaining a high index of suspicion for remote cosmetic procedures when evaluating deep‐seated gluteal masses that emerge long after a period of clinical dormancy.

Despite these clinical similarities, our case presents a profound histological divergence from established reports of PAAG complications. Historically, PAAG tissue reactions manifest in distinct histopathological forms: Christensen et al. [7] described unreactive PAAG as eliciting minimal host response, while inflammatory nodular reactions presented with an enhanced foreign body reaction and low‐grade bacterial infection within the first year postinjection. Alijotas Reig et al. [9] documented delayed immune‐mediated reactions following Aquamid administration, characterized microscopically by extracellular pools of gel, granulomatous inflammation, foreign body giant cells, and chronic inflammatory infiltration. The key novelty of our case resides in the specific intracellular architectural pattern, where the endocytosed Argiform material formed fine univacuolated and multivacuolated cytoplasmic spaces within histiocytes as nondigestible polymer. No extracellular material was identified. The absence of extracellular material is unlikely to be an artifact of tissue processing washout, given that no foreign body giant cell reaction was observed. This precise morphological pattern created a deceptive mimicry of signet‐ring‐like and multivacuolated lipoblasts, presenting a significant diagnostic pitfall for an ALT/well‐differentiated liposarcoma (WDLPS) [10]. While liquid silicone is well known for producing a “Swiss cheese” vacuolated pattern that mimics liposarcoma [11], this specific intracellular, lipoblast‐like histiocytic reaction secondary to Argiform has not been previously characterized. On routine light microscopy and IHC, the histopathological pattern of vacuolated, CD68‐positive histiocytic infiltrates remains nonspecific and cannot reliably distinguish PAAG from silicone or other permanent exogenous substances. Consequently, definitive identification of Argiform as the precise etiologic agent relied on targeted clinicopathologic correlation following the patient’s subsequent disclosure of her cosmetic injection history, rather than distinct light‐microscopic criteria alone.

We acknowledge that a punch biopsy has inherent sampling limitations compared to an excisional biopsy, as it captures a restricted tissue volume that may not fully reflect overall architectural patterns, depth, or peripheral tissue changes. However, in our case, the punch biopsy successfully yielded diagnostic tissue featuring characteristic intracellular histiocytic vacuolation. While excisional biopsy offers superior topographical assessment, punch biopsy provides a sufficient, minimally invasive method to establish the cellular pathology.

Infectious etiologies generating clear or vacuolated histiocytes, including atypical mycobacterial infections such as Mycobacterium avium complex and fungal infections such as *Histoplasma capsulatum*, were ruled out by negative AFB and GMS special stains. Noninfectious mimics were also excluded based on distinct morphological and clinical parameters: xanthomas typically exhibit uniform foamy cytoplasm rather than discrete vacuolation, Erdheim Chester disease demonstrates characteristic systemic involvement with Touton giant cells, and Langerhans cell histiocytosis displays grooved coffee bean nuclei with eosinophilic infiltrates [12]. Storage disorders such as Niemann Pick disease were clinically incompatible given the adult onset and isolated anatomical distribution.

IHC is indispensable in resolving the differential diagnosis in this case. True lipomatous tumors, including ALT/WDLPS, classically express S100 and display MDM2/CDK4 nuclear positivity or MDM2 gene amplification via fluorescence in situ hybridization (FISH) [13]. In our patient, the vacuolated cells were S100‐negative and CD68‐positive, definitively identifying them as histiocytes that had endocytosed exogenous foreign material rather than neoplastic adipocytes. Because the complete lack of S100 expression effectively ruled out an adipocytic proliferation, auxiliary MDM2 status testing was deemed unnecessary. Furthermore, negative pancytokeratin expression ruled out metastatic signet‐ring cell carcinoma. This diagnostic sequence highlights how a targeted IHC panel (CD68/S100), integrated with clinical history, efficiently excludes malignancy without requiring routine molecular testing. Additionally, our findings underscore the necessity of a high index of clinical suspicion and rigorous clinicopathologic correlation when encountering refractory plaques with unusual histology [1, 14].

Several treatment options are proposed with variable success rates depending on the nature of the cosmetic agent. Because resorbable fillers are readily degraded by the host, the first choice of treatment for foreign body granulomas caused by these agents is either systemic or intralesional corticosteroid injections [1, 2, 6, 15, 16]. In contrast, while permanent fillers are generally considered more challenging to address and surgical intervention remains the primary treatment option [1, 2, 17], conservative medical management with intralesional corticosteroid administration, such as triamcinolone acetonide, is frequently attempted as a first‐line alternative to minimize surgical morbidity, particularly in anatomically complex regions like the gluteal area. Corticosteroids work by suppressing local inflammatory cascades, reducing fibroblast proliferation, and dampening the active foreign‐body histiocytic response. In our patient, a targeted conservative regimen consisting of a single intralesional injection of triamcinolone acetonide (1.0 mL, 10 mg/mL) into the superior right buttock was selected to mitigate localized inflammation while avoiding the extensive tissue defects and scarring associated with aggressive surgical debulking. Although the procedure was well tolerated, loss to outpatient follow‐up remains a recognized limitation in managing postcosmetic complications. This lack of longitudinal evaluation highlights the broader clinical challenge in establishing the durable efficacy of nonsurgical interventions for permanent hydrogel reactions and tracking long‐term outcomes, such as symptom recurrence or secondary tissue atrophy.

In summary, while PAAG and its delayed latency are recognized features of complications, the novelty of this report lies in demonstrating that Argiform can induce an intracellular, vacuolated histiocytic response that closely mimics an ALT in the buttocks. Recognizing this specific histologic variant and utilizing IHC (S100 negative/CD68 positive) is essential to avoid misdiagnosing a benign foreign body reaction as a primary mesenchymal malignancy.

Declaration statement: Preliminary findings of this case have been reported in abstract form.

## Author Contributions

Mokhtar H. Abdelhammed and Lois M. Dodson contributed to writing the manuscript, and A. Hafeez Diwan and Ya Xu supervised the work.

## Funding

This study was not supported by any sponsor or funder.

## Disclosure

The preliminary findings of the manuscript were presented at the 2024 Trainee Research Symposium (Department of Pathology and Immunology, Baylor College of Medicine, Houston, TX, USA) and the 27th Joint Meeting of the International Society of Dermatopathology (March 6‐7, 2024, San Diego, CA, USA). All authors have read and approved the final manuscript.

## Ethics Statement

This retrospective review of patient data did not require ethical approval in accordance with local/national guidelines.

## Consent

No written consent has been obtained from the patient as there are no patient identifiable data included in this case report.

## Conflicts of Interest

The authors declare no conflicts of interest.
